# Can case-based discussions in a group setting be used to assess residents' clinical skills?

**DOI:** 10.5116/ijme.606a.eb39

**Published:** 2021-04-09

**Authors:** Rakel F. Johansen, René B. Nielsen, Bente V. Malling, Hanne Storm

**Affiliations:** 1 Steno Diabetes Center Aarhus, Aarhus University Hospital, Denmark; 2 DEFACTUM, Koncern Kvalitet, Central Denmark Region, Denmark; 3 Department of Clinical Medicine, Health, Aarhus University, Denmark; 4 Diagnostic Center, Regional Hospital Silkeborg, Regional Hospital Central, Jutland, Denmark

**Keywords:** Workplace-based assessment, case-based discussion, residents, internal medicine, clinical skills

## Abstract

**Objectives:**

The purpose of this study was to explore residents' and assessors' perception of a new group assessment concept.

**Methods:**

This qualitative study consists of observations of four group assessment sessions, followed by semi-structured interviews with six residents and four assessors (specialists in internal medicine), who all volunteered to be interviewed. All residents at a medical department (eleven to fifteen each time) and four assessors participated in four group assessments, where the residents' clinical skills were assessed through case-based discussions. An external consultant (an anthropologist) performed the observations and the interviews. Notes from the observations and the interviews were analyzed using an inductive approach.

**Results:**

Eight of the ten interviewed participants preferred group assessment to individual assessment. Results from the interviews suggested that the group assessments were more consistent and that the level of discussion was perceived to be higher in the group discussions compared to the one-to-one discussions. All residents indicated that they had acquired new knowledge during their assessment and reported having learned from listening to the assessment of their peers. Assessors similarly reported gaining new knowledge.

**Conclusions:**

The residents and assessors expressed very favourable attitudes toward the new group assessment concept. The assessment process was perceived to be higher in quality and more consistent, contributing to learning for all participating doctors in the department. Group assessment is feasible and acceptable, and provides a promising tool for assessment of clinical skills in the future.

## Introduction

Along with the implementation of competency-based education, workplace-based assessment (WPBA) has been widely adopted in many countries.^1^^-^^4^ Although the validity and reliability of WPBA methods has been discussed, evidence for their utility seems well-established for a number of assessment methods including case-based discussions (CbDs).^5^^-^^8^ Case-based discussions are well suited to assess trainees' clinical reasoning, decision-making and application of medical knowledge in patient care.^6^^-^^9^ The assessments also contribute to learning, especially if the assessments include formative feedback.^8^^,^^10^ Thus, many specialties have included CbD in their assessment program.^5^^,^^7^^,^^8^^,^^11^

The validity of WPBAs, including CbDs, is increased by choosing assessors who are most fit to judge the performance^12^; their value also depends on trainer enthusiasm^13^^,^^14^ and the training of the assessors.^15^^-^^21^ Assessor training is especially important for the assessment to be perceived as more than a tick-box exercise by both trainees and assessors.^6^^,^^7^^,^^14^^,^^15^^,^^22^^,^^23^ The reliability of CbD also increases with the number of assessments and assessors involved.^5^^,^^24^ Furthermore, the involvement of multiple assessors may lead to fairer assessment, due to decreased risk of bias that could be caused by personal relations.^16^

However, using many assessments and assessors might not be possible in a busy clinical environment even if the organization supports the use of WPBA. Implementation of CbDs and other WPBA methods needs to be feasible, as it takes a practical approach and involves the users.^6^^,^^12^^,^^17^^-^^20^

Organizational support is necessary to ensure that WPBAs are not just perceived as an additional workload and that they are used for relevant rather than easily assessable training activities.^19^

Most trainees appreciate the educational value of CbDs. However, more emphasis on the planning of these assessments is required.^20^^,^^24^^,^^27^^,^^33^ It has been suggested that the educational value and content of CbDs could be improved by providing ring-fenced time for these in both specialists' and residents' job plans to ensure sufficient time for the assessments.^7^^,^^11^^,^^13^^,^^21^^,^^23^

### Educational setting

In Denmark, which is the setting of this study, outcome-based education with mandatory WPBA, based on the seven CanMEDS roles, was implemented in 2004. In order to become a specialist, a doctor must go through a 2-year internship followed by four or five years of specialty training. All residents have an educational supervisor appointed for each rotation who is responsible for the resident's educational program and the assessment of their clinical skills in that particular rotation. One-to-one CbDs between resident and supervisor are a mandatory part of the evaluation of residents' clinicals skills.

### Rationale and study aim

Drawing on knowledge of the factors that increase the effectiveness of WPBAs, and in particular CbDs, the idea for a new "group assessment" concept took shape. The group assessment concept was created to increase the quality of CbDs used for assessing residents` clinical skills, and to make sure that the assessments were performed in a manner that was time- and cost-efficient.

In a medical department, mandatory one-to-one CbD assessments between the resident and the supervisor were therefore experimentally replaced by quarterly group assessment sessions. The aim of this study was to explore the residents' and assessors' perception of the new group assessment concept.

## Methods

### Study design

This study used a qualitative design consisting of observation of group assessment sessions, followed by semi-structured interviews with four specialists and six residents.

### Participants and setting

The study took place in a large multidisciplinary medical department. Group assessments were performed every third month over a year (four times in total), where every session lasted for 5 hours. All residents at the medical department participated (eleven to fifteen residents each time) as participation was mandatory. The residents were all five years or less from becoming medical specialists in rheumatology, endocrinology, cardiology, gastroenterology, or pulmonology. Four assessors and a moderator, all of whom were medical specialists, participated in each group assessment. Author HS served as a moderator at all of the sessions. An anthropologist (author RBN) observed the sessions and performed the interviews.

The study did not need formal ethics approval according to Danish law (Act on Research Ethics Review of Health Research Projects).^25^ However, all residents and specialists who volunteered to be interviewed received oral information about the study and gave verbal consent to participate, in agreement with national guidelines.^25^ Only the anthropologist had access to the interview data, and the participants were anonymized by designating them as resident 1-6 and assessor 1-4.

The schedule for the group assessments was sent out a year in advance; and three months before every meeting, the residents were reminded about the 3-4 predefined topics of the forthcoming session. The assessment group, who were medical specialists within the topic for the next session, were also invited to participate (Appendix 1).The group assessment sessions were scheduled and booked in the doctors' job plans.

The sessions took place in the departments' conference room with IT solutions available to present patient data. On the day of the group assessment, residents took turns presenting a clinical case within each of the day's topics (approximately 1 hour discussion for each topic), followed by a short discussion between the moderator and the assessors after each topic where the residents' performance was assessed.

All of the residents and specialists who participated in the sessions were later invited for interview; six residents and four assessors volunteered.

### Data collection

The data included two separate components: anthropologist observations and semi-structured interviews. The anthropologist observed the group assessment three times. The observations included observation of the structure and process, number of cases, the number of questions and comments, the placement of the participants, conversations, use of mobile phones/iPads, length of breaks and so on. The data from observations are field notes.

On the basis of observation of the first group assessment, the anthropologist developed an interview guide (Appendix 2). Thirty-minute semi-structured interviews using the interview guide were carried out. The interviews included topics such as experience with and attitude towards group assessment, comparison/experience with one-to-one assessment, the structure of the group assessment, preparation, outcome, case presentation and more.

All of the interviews and field notes were transcribed verbatim. The data were thematically categorized^26^ according to the interview guide by the author RBN, who read the material and identified main themes and sub-themes.

**Table 1:** Interview themes and quotations

Theme	Residents	Assessors
Overall perception of group assessment	"I think it is a much better idea than the way it works other places. We have to have these signatures…." (resident 4) "I think it is a very big relief to have your competences approved in this manner, because the other way [one-to-one assessment] is chaotic and random." (resident 6)	"After participation, I felt very positive about the concept. I really think it was very rewarding in many ways. It was definitely something that gave new dimensions that I have not found in other places…" (assessor 1)
Group assessment versus one-to-one assessment	"There are advantages and disadvantages related to both types of assessment, but I think I would personally benefit most from the one-to-one assessment. I have nothing negative to say about group assessment. I think [group assessment] is a good way to have your competences approved." (resident 2) "It is academically very enriching that many fields of medicine are gathered. We come together and discuss – which is the opposite of what my fellow residents experience in other places. I have previously participated in one-to-one assessments where you just meet with your supervisor and look at a couple of medical records and then the supervisor says: "It looks good" and there is no more discussion.… So, I would definitely say that it is worthwhile to use 5 hours 4 times a year on group assessments where many provide me with feedback." (resident 4)	"One-to-one assessments are very shallow. It is a bit like: "Well, this looks good. Do you have any comments?" and "We have to have this signed". There was very little content in the one-to-one assessment and it was done because we had to do it, not in order to achieve anything." (assessor 1)
Outcome regarding learning, motivation and attitude	"Well, the good thing is that we come from different fields of medicine. This is valuable as if you, for instance, present a case with a rheumatologic disease, then it is exciting to hear what the doctors in the field of rheumatology have to say about the case." (resident 5) "I look at it as a good opportunity to discuss common challenges. I mean certain types of patients and illnesses. So, I see it more as an opportunity for discussion than an assessment of my competences." (resident 4)	"….I am, for instance, a specialist in gastroenterology and I supervise a resident, but I also have to approve competences that are outside the field I normally deal with." (assessor 3) "I think it works well; you get through a lot of topics during group assessment…." (assessor 3)
Context and structure regarding preparation, time used, case presentation and the moderator	"Group assessments are announced well ahead of time. You know what the topics are so you pay attention to them in your daily clinical work – if you encounter something exciting. You want to find a case with substance that you think is interesting for your colleagues to hear about." (resident 5) "I think the length of the session was very suitable. You can feel a bit tired at the end, but I don't think it is a problem. I have tried to attend something for just five minutes where I have been more tired. It is, after all, a matter of how interesting it is." (resident 4) "There has been a tendency to pick good and exciting cases. However, in our field, we could be better at presenting what is difficult or unclear, because that is what we often encounter in our daily clinical work…" (resident 1) "The cases that are presented are cases that were challenging and difficult. …… It has to be a case where you were challenged while on night shift or on the ward round or something else, where you had a hard time solving the problem, and then you can present this issue." (resident 2) "The moderator makes sure that everyone gets to present and contribute to the discussion, and this ensures that everyone is well prepared for the session." (resident 4)	"The second time I participated, it worked out really well. ……... The residents all came well prepared and we did too. Everyone was set on getting a really good discussion of the cases." (assessor 2) "I have been very positively surprised about how well prepared the residents have been, also with regard to background knowledge…" (assessor 4) "We talked a lot about how important it is to have someone to steer the meeting and that the moderator makes sure everyone has contributed to the discussion, or when it is necessary to confront residents who have been less active in the discussion." (assessor 4) "I think the whole concept depends on a really good moderator who can steer the meeting and make sure that the purpose of the group assessment is met, which is that all residents get their competences approved." (assessor 4)
Relation	"When you come to a new department and join the group assessment, it is also a good place for social networking, because you have five hours together…. you are in dialogue with the others and feel safe." (resident 1)	

The categorization, themes and sub-themes were discussed with author HS until consensus was reached. Qualitative guidelines were followed to ensure transparency.^27^^,^^28^ Quotations that most accurately illustrated the sub-themes were selected and translated from Danish into English. Table 1 shows the themes and sub-themes with examples of statements belonging to the themes.

## Results

Observations of the group assessment are presented, followed by findings from the interview data which are divided into four major themes.

### Observation of the group assessment

Clinical cases were discussed with both peers and the assessors. The moderator kept track of time and made sure that all residents participated in the group discussion and the presentation of cases. The four assessors and moderator together assessed the residents' skills, and feedback was given during the discussion of the case. Notes and mandatory standard assessment forms were used for the assessment.

The anthropologist could not evaluate the medical content of the discussion, but a case could result in a discussion of many other aspects than the core medical content of the case, such as social background, ethics, and communication. This generated lots of input from both residents and assessors. Many of the presented cases were known by several of the doctors, who could provide different perspectives. The number of doctors who took part in the discussion after each case presentation varied considerably, and there was also great variation in the amount of time used on each case discussion (from 2 to 20 minutes). The assessors were also active to varying degrees, especially with respect to follow-up questions for assessment of satisfactory clinical competencies of the resident, suggesting a different understanding of the role of assessor, which was confirmed during the interviews.

It was noticed that the moderator played a powerful role in ensuring that all topics were discussed and that all residents participated in the case presentations and subsequent discussions.

After each topic, the assessors and the moderator discussed in private the performance of the residents and came to a consensus on whether their competence should be approved. If a resident did not perform sufficiently, a one-to-one assessment together with the educational supervisor was arranged. The results of the assessment were communicated to both the resident and the educational supervisor at a later point in time to avoid humiliating the resident in a group forum.

### Interview data

### Preparing for the group assessment and case presentation

The residents felt that they had plenty of time to gather and prepare cases, as the topics and dates for the group assessments were sent out a year in advance, with the following reminder three months prior to the next group assessment:

"You know what the topics are, so bear this in mind if you encounter an interesting patient. You want to find a case with many interesting aspects that you think will be of interest to all your colleagues." (resident 5)

The residents remembered the cases by making notes about them and reading the notes again before the group assessment. The assessors were a bit nervous about what to expect prior to the first group assessment, but found they did not really need to prepare:

" The first time I was unsure about what to expect, but I think it went well… I found out that I knew the things I should know, and it did not require much preparation." (assessor 3)

The residents themselves selected the cases they wanted to present. As some pointed out, this could result in them choosing cases where they felt confident about their own knowledge. Several residents stated that there might be a tendency to pick out cases that were rare and intriguing:

"There has been a tendency to pick good and exciting cases. However, in our field we should be better at presenting what is difficult or unclear, because that is what we often encounter in our daily clinical work…." (resident 1)

Other residents, on the contrary, stated that they were more likely to present difficult cases where they had been unsure about what to do. Yet another resident stated that she chose to present a case within her own specialty that she thought was important for all doctors to have some knowledge about.

### Assessment, resources, and approval of competences

It was often stated that it could be difficult to assess all of the residents at the same time. Someone might hide in a group, not necessarily because he/she lacked the required competences. This could be due to their personality, as some are more introvert and reserved, while others are extrovert and like to be heard:

"You are in a forum, where one might feel a bit exposed. You are together with specialists from the department, and everybody has that basic fear: one does not want to present oneself as professionally ignorant." (resident 1)

Many doctors acknowledged the ability of the moderator to steer the meeting and in ensuring everyone was heard. The length of the group assessment was regarded as suitable and many emphasized that the sessions were relaxed with a good and safe environment that was not exam-like. Since there were several assessors, many found that the assessment was more consistent. Although it took time to plan the group assessments, both the residents and assessors felt that they were more resource-efficient and manageable than one-to-one assessments.

As the meetings were scheduled during working hours, it was highly appreciated that group assessment made approving competences straightforward. One resident pointed out that previously having competences approved often became a hunt for signatures, without proper assessment or feedback:

 " I think this is much better than the way it works in other places. We have to have these signatures, so many signatures… It is a bit of a hunt for signatures, without anyone really going into the depth with anything." (resident 4)

### Professionalism, learning and interdisciplinarity

The residents especially appreciated professional discussions in an interdisciplinary environment. They found it professionally enriching and educational to meet with doctors from other specialties to discuss common challenges. They also mentioned that the group assessments provided insights into the other doctors' skills that could inspire them to become just as skilled:

"You get a better insight into the other doctors' skills, and this can serve as inspiration to become just as skilled as them." (resident 5)

For the assessors, the assessment process had a greater focus than the professional discussions, but they also acknowledged that the doctors gained more knowledge from the group assessments than the one-to-one assessments. The extra time available to discuss cases was greatly appreciated. As one assessor put it:

 "…. I think we all felt it was a luxury to have time for discussion. Because we all learn from each other, no matter if you are a resident or an expert. But the expert is not an expert in all medical fields, so in that way it is mutually beneficial, although the assessment is about them [the residents]. As such, I think they get greater knowledge than in the one-to-one assessments." (assessor 2)

Both residents and assessors thought that group assessments were rewarding for all. Most participants looked forward to the next group assessment, because they gained new knowledge and insights during the sessions. Group assessments made it possible to discuss more cases (around 24 cases in a session) compared to the number of cases covered at the one-to-one assessments (usually three to four). Furthermore, the presence of residents and assessors from different specialties provided more perspectives and interdisciplinary knowledge. One resident thought the concept was a stroke of genius:

" The basic idea to meet four times a year and have competences assessed in a forum where residents and specialists are gathered is an excellent idea. It shows that this is something that needs to be done, and there is a setting and a deadline for the assessment." (resident 1)

### Overall perspective on the group assessment concept

Eight of the ten doctors who were interviewed preferred group assessment to the one-to-one CbDs, while two residents preferred the individual one-to-one assessments, provided there was enough time for case discussion;

"I think I would personally benefit most from the one-to-one assessment. But it [group assessment] is a good way to have your competences assessed." (resident 2)

The residents acknowledged the value of individual supervision received from their educational supervisor, but for the approval of core internal medicine competences, group assessment was considered superior by most.

Many of the doctors therefore suggested that group assessment should be tried out and subsequently implemented at other departments, to replace the mandatory one-to-one CbD assessments of key competences.

## Discussion

By introducing group assessment, it seems that some of the obstacles associated with one-to-one CbDs for assessment of clinical competences can be overcome. By including many assessors, it is likely that there is less subjectivity and bias in the assessment process^16^; additionally, specialists who are best suited to assess particular competences are asked to participate.^12^ Furthermore, including assessors and residents from different specialties raises the level of discussion, and gives a broader perspective on the cases and required skills. For instance, the emphasis may be quite different when it comes to a discussion of "dizziness" if you are a cardiologist or a geriatrician.

The assessors understood their role as assessors differently, so perhaps using assessors who were already familiar with the concept could have improved the group assessment.^29^ However, discussions among the assessors regarding whether or not a resident had the necessary competence to get approval contributed to the assessor's knowledge and skills as assessors and led to a common understanding of the concept of CbD, and how it was to be used in this specific department. The need to be trained as an assessor has been recommended by several authors.^15^^,^^30^^,^^31^ The introduction of this group assessment concept thus provided a learning opportunity for the assessors regarding the use of CbD. Whether this led to an increase in the quality of other assessments in the department was beyond the scope of this study. However, the general agreement on the pass/fail level obtained through discussions among the assessors does lead to higher uniformity and probably makes the assessments fairer.

The residents themselves chose the cases that they wanted to present. As pointed out by the residents, they might present cases where they had performed well, so CbDs alone might not give a reliable picture of a resident's competences. However, the interviews showed that residents also chose cases where they had been in doubt. Further studies are needed to answer the question of how residents choose cases for CbDs.

One of the difficulties reported by the assessors was the variation in the residents' contribution to the discussions, which might lead to more introvert residents being overlooked. All assessors stressed the importance of the moderator in making sure all residents were active. Besides this, the moderator should steer the conversation and ensure group effectiveness. These are all competences ascribed to a good facilitator.^32^ In the opinion of the assessors, the moderator plays a crucial role in the group assessments' success.

The group assessment concept provided ring-fenced time for the assessment of residents' clinical skills. Dedicating 4x5 hours for group assessment and gathering many of the doctors in the department may seem an unaffordable solution. It requires planning to ensure that residents and specialists are not scheduled to do other tasks, and that the residents have enough time to find cases. This requires an open-minded head of department, willing to invest time on education. However, the investment seems to be worthwhile, as initiatives like these increase attention on specialty training and place specialty training on the agenda of clinical departments.^19^ Thus, the introduction and implementation of group assessments is an example of how new initiatives in health care can succeed if local solutions are accepted. It might reflect the freedom in interpreting and implementing competency-based medical education to make it fit locally, as recently called for by Dagnone and colleagues.^20^

To our knowledge, similar studies regarding group assessment of residents` clinical skills have not been made, but the concept may resemble practice-based small group learning (PBSGL) where groups of doctors gather to discuss cases from daily clinical work, and case presentations are often followed by topic review and discussions of the related evidence-based publications to identify implications for changes in practice.^33^ The concept is widely used by general practitioners and seems to be a promising method of continuing professional development.^33^ In this study, it was found that group assessments enhanced the learning experience of both residents and assessors and may also have contributed to continued professional development of the assessors.

### Limitations

A limitation of the study was that residents and assessors volunteered for the interview, and so doctors who were either very fond of or dissatisfied with group assessment might be more likely to volunteer. This might have given either more positive or more negative results. However, there were both positive and negative evaluations expressed in the interviews. Thus, the results seem to reflect reality.

Another limitation is that only one of the authors of this paper performed the data analysis. However, the categories and themes were discussed and agreed by two of the authors (HS, RBN), who also attended all the group assessment sessions.

Furthermore, it is difficult to consider the generalizability of the results from this study, since only one department participated. It might not be possible to implement group assessment in all departments or in all specialties. However, most specialties have general competencies and many specialties use CbD in their assessment program, and, as such, it might be interesting to try implementing the concept in other specialties and departments.

## Conclusions

The group assessment concept seems to offer an acceptable, feasible and efficient model for CbD used as a formative assessment of residents' competences in internal medicine. It reduces the effect of interpersonal relations between residents and supervisors and thereby minimizes bias. It provides busy clinicians with time to engage in teaching/assessment activities. The amount of knowledge, skills, input and inspiration grows with the number of residents and medical specialists. Thus, group assessment serves as a tool for assessing clinical skills, and facilitates learning for all of the participating doctors in the department.

The group assessment concept with the goal of assessing residents' competences, together with mutual learning, could serve as an inspiration for other departments, specialties, and countries. Further studies are required to investigate the value of group assessment as a tool for assessing doctors' clinical skills.

### Acknowledgments

We would like to thank all of the doctors who participated in the group assessments, and, in particular, the doctors who volunteered in the interviews. We would also like to thank the head of the Medical Department, Diagnostic Center, Silkeborg for their openmindedness in trying out a new concept.

### Conflict of Interest

The authors declare that they have no conflicts of interest.

## Supplementary material

Supplementary file 1 Appendix 1. Topics discussed every third month with participating residents and assessment group specialists

Supplementary file 2 Appendix 2. Interview guide
