# Supplementary material for: Can case-based discussions in a group setting be used to assess residents' clinical skills?
Source: Int J Med Educ. 2021 Apr 9;12:64–73. doi: 10.5116/ijme.606a.eb39 (PMC8411343; doi:10.5116/ijme.606a.eb39)
Supplement: Supplementary file 1 — Appendix 1. Topics discussed every third month with participating residents and assessment group specialists [file ijme-12-64-S1.pdf]

Appendix 1.

Topics discussed every third month with participating residents and assessment group specialists

Internal Medicine group assessment	Topics for case-based discussions	Assessment group specialists in:
March	Chest pain<br>Dyspnea<br>Edema<br>The shocked patient	Cardiology<br>Pulmonology<br>Internal medicine<br>Internal medicine
June	Fever<br>Weight loss<br>Musculoskeletal pain<br>Blood disorders	Infectious diseases<br>Gastroenterology<br>Rheumatology<br>Haematology
October	Stomach pain, diarrhea and obstipation<br>Water-electrolyte and acid-base imbalances	Gastroenterology<br>Endocrinology<br>Nephrology<br>Internal medicine
December	Dizziness and falls<br>Terminal illness<br>Poisoning	Geriatrics<br>Endocrinology<br>Cardiology<br>Internal medicine
