# Supplementary material for: Can case-based discussions in a group setting be used to assess residents' clinical skills?
Source: Int J Med Educ. 2021 Apr 9;12:64–73. doi: 10.5116/ijme.606a.eb39 (PMC8411343; doi:10.5116/ijme.606a.eb39)
Supplement: Supplementary file 2 — Appendix 2. Interview guide [file ijme-12-64-S2.pdf]

Appendix 2.

Interview guide

**Residents**		
First/last part of the residency:	Number of times attending the group assessment:	
Experience and attitude towards workplace-based assessments and case-based discussions		Experience with workplace-based assessments and case-based discussions at medical school, during medical training, and in other/previous settings.<br>What do you think about the group assessment?<br>How does this method of competence assessment suit your personality?
Group assessment		What do you gain from group assessment?<br>Do you learn from group assessment? (Please give examples)<br>Does knowledge of other residents and specialists motivate you to seek more knowledge yourself?<br>What is the attitude toward group assessment of your colleagues who do not participate in the group assessments?
Comparison with individual one-to-one assessment		What was the difference?<br>What was good?<br>What was bad?<br>If you had to choose – what would you prefer: group assessment or individual one-to-one assessment?
Context		Do you prepare yourself? (How much?)<br>Length of session – is the session too long or too short?<br>Case presentation – does it work?<br>The moderator – is everyone heard (is it possible to hide?)<br>How is the assessment performed?<br>Is group assessment perceived to be stressful?<br>Is it "easy", for instance, to say "I don't know"?
Elements/parts		What elements of group assessment are most important?<br>Are there elements that could be omitted?
Subsequently		Have group assessments changed your cooperation with colleagues/other residents?<br>Do you think that group assessment should be changed in any way?<br>Do you think the group assessment concept could be used in other specialties/departments?
Department reputation		Is there a link between the reputation of the department and the performance of group assessments?<br>What could profitably be changed?
**Specialists/supervisors**		
Your immediate perception of the group assessment concept?		Is group assessment the solution to the problem it was intended to solve?<br>Does group assessment save time compared to the individual one-to-one assessment?<br>In what way do specialists profit from group assessment?
Context		What do you think of group assessment?<br>Is the meeting too long or too short?

	The moderator – how does it work?<br>The assessment – how does it work?<br>Does the assessment in itself work?<br>Are the residents well prepared, in your opinion?<br>How do you ensure that all of the residents participate actively in the discussion?
Elements/parts	If group assessment is split into parts, what parts does it consist of?<br>What elements concerning group assessment are most important?
The concept	Do you think that group assessment could be used in other specialties/departments?<br>Advantages/disadvantages?<br>In your opinion, what could profitably be changed?
Department reputation	Is there a connection between the reputation of the department and group assessment?
